# Normal liver stiffness values measured with MR elastography and two-dimensional shear wave elastography in children aged 9–17 years

**DOI:** 10.1007/s00261-026-05431-2

**Published:** 2026-04-27

**Authors:** Mehmet Seker, Mehmet Sait Dogan, Hayrettin Temel, Osman Kahraman, Fatma Zeynep Gungoren, Mehmet Kocak, Cengiz Erol

**Affiliations:** 1 Department of Radiology, Istanbul Medipol University, School of Medicine, Istanbul, Turkey; 2 Department of Pediatrics, Istanbul Medipol University, School of Medicine, Istanbul, Turkey; 3 Department of Radiology, Anadolu Medical Center In Affiliation with Johns Hopkins Medicine, Istanbul, Turkey; 4 Department of Biostatistics, Istanbul Medipol University, School of Medicine, Istanbul, Turkey

**Keywords:** Magnetic resonance elastography, Ultrasound elastography, Liver stiffness measurement, Healthy children

## Abstract

**Purpose:**

This study aimed to establish pediatric reference ranges for liver stiffness measured by magnetic resonance elastography (MRE) and two‑dimensional shear‑wave ultrasound elastography (2D‑SWE) in healthy Turkish children aged 9–17 years.

**Materials and methods:**

Of the 100 initially recruited volunteers, nine were excluded based on strict imaging and biochemical criteria. A final cohort of 91 healthy participants with normal clinical, biochemical, and serological evaluation underwent same‑day MRE (3.0 T, external 60 Hz pneumatic active driver, anterior passive driver paddle over the right upper quadrant, breath‑hold acquisition; non‑vascular ROIs in the right lobe) and 2D‑SWE (intercostal approach, supine position, right arm elevated, 3–5 circular ROIs placed 1.5–2.0 cm below the liver capsule in the right lobe, during end‑expiration breath‑hold). Reference intervals were calculated as mean ±1.96 SD and represent modality‑specific reference limits. Group comparisons were evaluated using the Wilcoxon–Mann–Whitney and Kruskal–Wallis tests.

**Results:**

Mean liver stiffness values (LSV) were 2.16 ± 0.24 kPa for MRE (reference interval 1.69–2.63 kPa) and 5.34 ± 1.00 kPa for 2D‑SWE (reference interval 3.38–7.30 kPa). No significant differences were observed across age groups (9–11, 12–14, 15–17 years) or between sexes. Correlations with laboratory values were limited and clinically non‑significant in this healthy cohort.

**Conclusion:**

Same‑day MRE and 2D‑SWE provide modality‑specific pediatric liver stiffness reference ranges in healthy children aged 9–17 years. These values appear independent of age and sex and should be interpreted as non‑interchangeable, modality‑dependent reference intervals.

## Introduction

The worldwide increase in obesity has led to a rising incidence of hepatic steatosis and related non-alcoholic fatty liver disease in children. Fatty liver disease and other chronic liver parenchymal diseases, such as hepatitis, can progress to fibrosis. If left untreated, fibrosis may lead to cirrhosis, neoplasia, and liver failure. The diagnosis and grading of fibrosis are important because fibrosis is now known to be reversible with early treatment [1–4].

Fibrosis reflects collagen/extracellular matrix accumulation after chronic injury. Although liver biopsy remains the reference standard, it is invasive, carries a risk of bleeding, and is limited by sampling error (approximately 14.5%) due to the small tissue volume obtained and the heterogeneous distribution of fibrosis [2, 5–7]. Accordingly, US- and MR elastography are increasingly used for diagnosis and longitudinal monitoring by noninvasively quantifying liver stiffness. Compared with biopsy (< 1 cm³), shear-wave US elastography (~20 cm³) and MRE (~250 cm³) assess substantially larger tissue volumes, thereby improving the evaluation of fibrosis severity.[6, 8].

Ultrasound (US) elastography includes strain- and shear-wave–based methods; in routine practice, shear-wave techniques (e.g., transient elastography and acoustic radiation force impulse–based SWE, including 2D‑SWE) are most commonly used [9, 10]. US-based liver stiffness measurements may be elevated in the absence of fibrosis (e.g., congestion/increased central venous pressure, cholestasis, and acute inflammation) and can vary with technique and acquisition conditions [11–13] Reported normal adult stiffness values are approximately 4.8–5.5 kPa [14, 15] ,while pediatric reference values may differ by age group and method.[16].

MRE generates externally driven mechanical waves and provides quantitative stiffness estimates, sampling a larger hepatic volume [2]. Measurement quality may be reduced by iron-related signal loss and elastogram reliability may decrease in advanced steatosis [1, 17–19]. Across studies, MRE reference values vary with population and protocol (including fasting status). Comparative normative data obtained with both MRE and 2D‑SWE on the same day in the same healthy pediatric cohort remain limited, and data specific to Turkish children are scarce. Accordingly, the primary objective of this study was to establish modality‑specific reference ranges for liver stiffness measured by same‑day MRE and 2D‑SWE in clinically and biochemically healthy Turkish children aged 9–17 years and to facilitate direct inter‑modality benchmarking under standardized conditions. Secondary analyses assessed differences by age group and sex, while correlations with laboratory values were performed as exploratory, hypothesis‑generating analyses.

## Materials and methods

This single-center, prospective observational study included 100 healthy volunteers aged 9–17 years with no known liver disease. Participants were divided into three age groups: 9–11, 12–14, and 15–17 years. Sex, height, weight, and body mass index (BMI) were recorded. Because splanchnic circulation after oral feeding may affect liver stiffness measurements, participants were instructed to fast for at least four hours before imaging. Screening biochemistry and viral hepatitis serologies were obtained prior to imaging. Eligibility required anthropometric, biochemical, serologic, and MRI-based screening findings. Specifically, participants were required to have a BMI percentile between the 10th and 95th percentiles (inclusive). Based on institutional pediatric reference ranges, liver biochemistry thresholds for inclusion were defined as follows: aspartate aminotransferase (AST) ≤ 60 U/L, alanine aminotransferase (ALT) ≤ 48 U/L, gamma-glutamyl transferase (GGT) ≤ 61 U/L, direct bilirubin ≤ 0.8 mg/dL, total bilirubin ≤ 1.5 mg/dL, and alkaline phosphatase (ALP) ≤ 500 U/L (to account for physiologic bone turnover in the adolescent age group). Any biochemical or serological finding indicating potential liver disease led to exclusion.

Viral hepatitis serologies were classified using signal-to-cutoff (S/CO) ratios, where < 0.9 was considered nonreactive and ≥ 1.0 reactive. Equivocal results (0.9 to < 1.0) were retested. Inclusion required nonreactive results for anti-HCV, HBsAg, and anti-HBc. For MRI-based inclusion, a proton density fat fraction (PDFF) ≤ 5.5% and no evidence of iron accumulation (R2* ≤ 126 s⁻¹) were required.

A total of nine participants were excluded based on the predefined criteria. Six participants were excluded due to MRI-determined hepatic fat fraction > 5.5% and ultrasonographic evidence of steatosis. One participant was excluded due to transient hepatic dysfunction, characterized by an elevation in liver enzymes that normalized upon repeat testing after clinical recovery. Another participant was excluded for persistent liver enzyme elevation secondary to chronic parenchymal liver disease. Finally, one participant was excluded due to claustrophobia, which prevented MR completion. These exclusions were implemented to maintain a healthy cohort and avoid confounding from hepatic dysfunction or metabolic abnormalities. Finally, 91 healthy participants with normal clinical, biochemical, and serological evaluation underwent same‑day MRE and 2D‑SWE.

In US elastography, all examinations were performed by a radiologist with 13 years of experience in abdominal ultrasound and elastography. The liver parenchyma was first evaluated for incidental focal lesions using B-mode US (LOGIQ P9, GE Healthcare, USA) using a convex transducer (1–6 MHz) with the abdomen preset and the participant in the supine position. Hepatic steatosis was graded qualitatively (grade 1–3) based on predefined sonographic features (hepatorenal echo contrast/bright liver for mild; additional vessel blurring for moderate; and additional posterior beam attenuation with reduced diaphragm visualization for severe steatosis) [20]. Each participant’s right hand was placed behind the head to widen the intercostal spaces. Using two-dimensional (2D) shear wave elastography, measurements were obtained from the right hepatic lobe via an intercostal approach during an end-expiration breath-hold. A circular ROI of 8–10 mm was placed 1.5–2.0 cm below the liver capsule, avoiding vascular structures and rib-shadow artifacts. 12 measurements were acquired, and the median liver stiffness value (kPa) was used for analysis. Measurements were considered acceptable when the interquartile range-to-median ratio (IQR/median) was < 30%.

In MR elastography (MRE), cases were evaluated with a 3 T MRI scanner (Magnetom Vida, Siemens, Erlangen, Germany) in the supine position. A phase-contrast echo-planar imaging (EPI) sequence with motion-encoding gradients was used at end-expiration together with an active driver (Resoundant, Rochester, USA) set to a frequency of 60 Hz [2, 17, 21–23]. A rigid passive driver was placed in the right midclavicular line at the level of the xiphoid process and positioned in full contact with the body surface in order to achieve optimal wave propagation [2, 24].

To prevent fold-over artifacts, the vibration amplitude was adjusted according to the patient’s weight [22, 23]. The amplitude was set to 10–20% for patients weighing 20–29 kg, 20–30% for those weighing 30–39 kg, 30–40% for those weighing 40–59 kg, 50% for those weighing 60–69 kg, and 60% for those weighing 70–85 kg. General anesthesia was not used during MRI. Each participant was trained to perform breath-holding at end-expiration, and the quality of the MRE examination was checked in all cases. The MRE acquisition parameters for the EPI sequence were as follows: slice thickness, 10 mm; number of slices, 4; slice gap, 0 mm; field of view (FOV), 200 mm; matrix, 256 × 64; repetition time (TR), 50 ms; flip angle, 20°; number of excitations (NEX), 1; phase-encoding direction, right-to-left; and acceleration, GRAPPA [24].

On magnitude images, signal loss in the subcutaneous fatty tissue at the location of the passive driver was used to confirm transmission of the mechanical waves. On phase images, the waveforms were examined to ensure that they progressed approximately parallel to the liver capsule. In healthy cases, the waves are expected not to be distorted and to gradually attenuate toward the center of the liver. Subsequently, elastograms and inversion-derived confidence/quality maps (confidence masks indicating regions with reliable shear-wave inversion) were generated [2, 17]. LSV (kPa) was calculated using the inversion algorithm, excluding pixels with low confidence (masked regions), by placing an ROI of at least 250 cm³ in the non-shaded, four different sectional area of the right hepatic lobe. The ROI was placed 1–2 cm away from the liver capsule [1, 5]. PDFF was derived using a six-echo single-breath-hold Dixon sequences. Iron accumulation was evaluated using R2* and R2 water maps [21–23]. All MR images were evaluated by a radiologist with 16 years of experience in abdominal MR and MRE.

We used both MRE and 2D-SWE to summarize liver stiffness measurements. Modality-specific reference intervals were calculated as mean ±1.96 standard deviations. To quantify uncertainty around these reference limits, 95% bootstrap confidence intervals for the lower and upper reference limits were estimated using nonparametric bootstrap resampling.

Normality of the liver stiffness measures was assessed using Shapiro-Wilk normality test for MRI and US-based measurements. For group comparisons across age strata and sex, we used a conservative nonparametric approach (Kruskal–Wallis and Wilcoxon–Mann–Whitney tests), given potential deviations from parametric assumptions and unequal variances across small strata. For example, to compare the distribution of a continuous variable across the levels of a categorical variable, we used the Wilcoxon–Mann–Whitney or Kruskal–Wallis tests, which are non-parametric counterparts of the Student t test and one-way ANOVA, respectively. Associations between continuous variables were evaluated using Spearman’s rank correlation coefficient and were considered exploratory. All tests were two-sided, and statistical significance was set at *p* < 0.05. Because p values were not adjusted for multiple comparisons, inferential results—particularly correlation analyses—should be interpreted as hypothesis-generating. Analyses were performed using SAS^®^ version 9.4 (Cary, North Carolina, USA).

## Results

### Study group and descriptive statistics

**Fig. 1:**
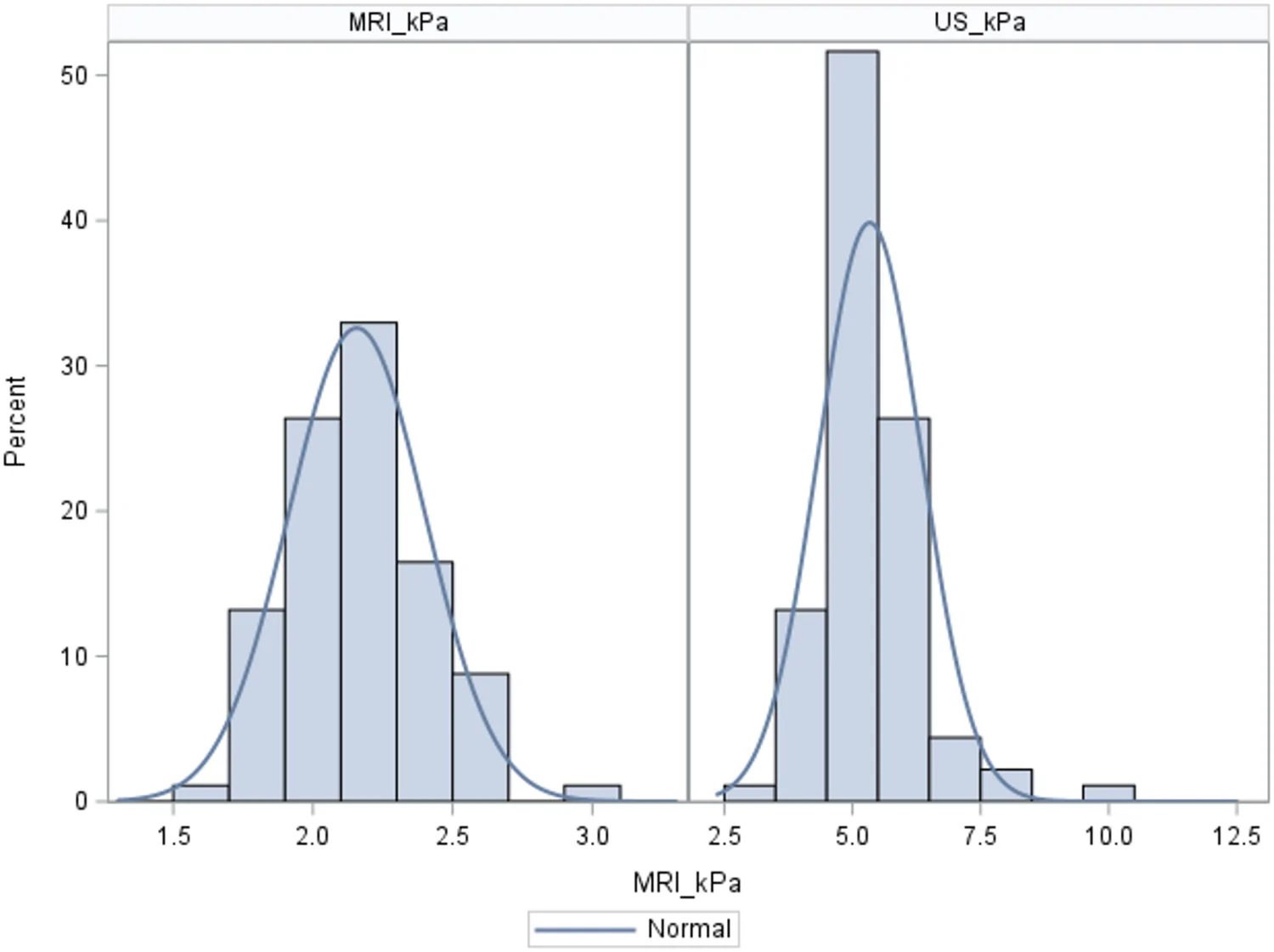
General distribution of MRI (MRI_kPa) and ultrasound (US_kPa) liver stiffness values, MRI_kPa and US_kPa represent liver stiffness values (kPa) measured by MR and US elastography, respectively

**Fig. 2:**
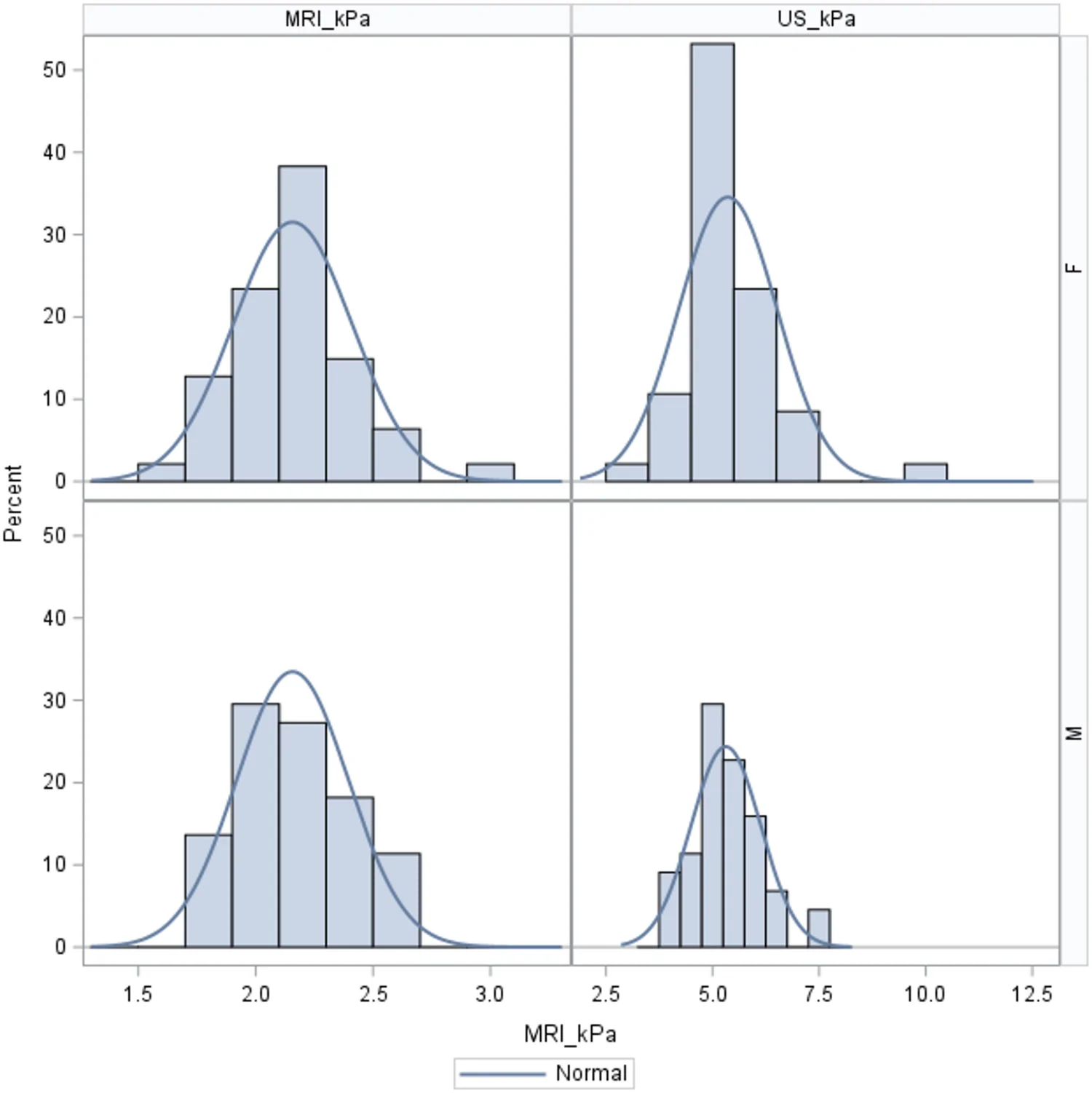
Distribution of liver MRI (MRI_kPa) and ultrasound (US_kPa) stiffness values by sex (F: female, M: male). MRI_kPa and US_kPa represent liver stiffness values (kPa) measured by MR and US elastography, respectively

Of 100 volunteers initially enrolled, 91 participants (9–17 years) were included in the final analysis. Nine participants were excluded for predefined reasons. Forty‑seven participants were female (51.7%) and 44 were male (48.3%). Participants were grouped by age (9–11, 12–14, 15–17 years) to reflect developmental stages and expected differences in breath‑hold performance; children younger than 8 years were not recruited because reliable breath‑holding for MRE acquisition was not feasible. Clinical, laboratory, and imaging characteristics are summarized in Table 1. Liver stiffness values are denoted as MRI_kPa for MRE and US_kPa for 2D-SWE. Distributional characteristics of MRI_kPa and US_kPa were assessed using the Shapiro–Wilk test (*p* = 0.99 and *p* = 0.89, respectively). The distributions of MRI_kPa and US_kPa were approximately symmetric (Figs. 1 and 2).

**Table 1:** Characteristics of the study population

	Min	Q1	Median	Q3	Max	Mean	SD
Age	9.00	11.00	13.00	15.00	17.00	12.85	2.65
Height	1.25	1.45	1.56	1.64	1.90	1.55	0.15
Weight	25.00	40.00	48.00	60.00	93.00	49.62	14.37
Body-mass index	14.27	17.86	20.01	22.6	34.58	20.33	3.54
MR fat fraction	0.30	1.40	2.00	2.80	5.40	2.18	1.04
R2*	1.90	31.00	34.00	37.4	45.00	33.25	7.16
R2 water	7.70	31.80	33.60	35.0	45.00	33.37	4.26
AST	12.00	18.00	21.00	26.0	60.00	23.20	8.87
ALT	6.00	11.00	12.00	17.0	39.00	14.33	6.08
GGT	5.00	9.00	10.00	12.0	26.00	10.86	3.47
ALP	17.00	86.00	199.00	255.0	459.00	184.77	98.96
Total bilirubin	0.10	0.28	0.40	0.55	1.48	0.45	0.26
Direct bilirubin	0.08	0.17	0.23	0.28	0.74	0.24	0.12
MR elastography (kPa)	1.61	2.00	2.16	2.30	2.90	2.16	0.24
US elastography (kPa)	3.07	4.82	5.23	5.78	10.38	5.34	1.00

Data are presented as median (Q1–Q3) unless otherwise specified. *Min* minimum, *Q1* first quartile (25th percentile), *Q3* third quartile (75th percentile), *Max* maximum, *SD* standard deviation

### Comparison of liver MR and US stiffness values with other parameters

Liver stiffness did not differ by sex for either MRE or 2D-SWE (Table 2). In exploratory analyses (Table 3), BMI showed a weak negative correlation with both MRI_kPa (*ρ* = −0.28, *p* = 0.0082) and US_kPa (*ρ* = −0.22, *p* = 0.039). According to linear regression models, the BMI slope for MRI_kPa was −0.015 kPa per BMI unit, which is considered clinically negligible. In contrast, BMI was not a statistically significant predictor of US_kPa (slope = 0.019 kPa per BMI unit, *p* = 0.53). Finally, MRI_kPa and US_kPa were positively associated (*ρ* = 0.34, *p* = 0.0011).

### Liver MR and US stiffness values by age group

**Fig. 3:**
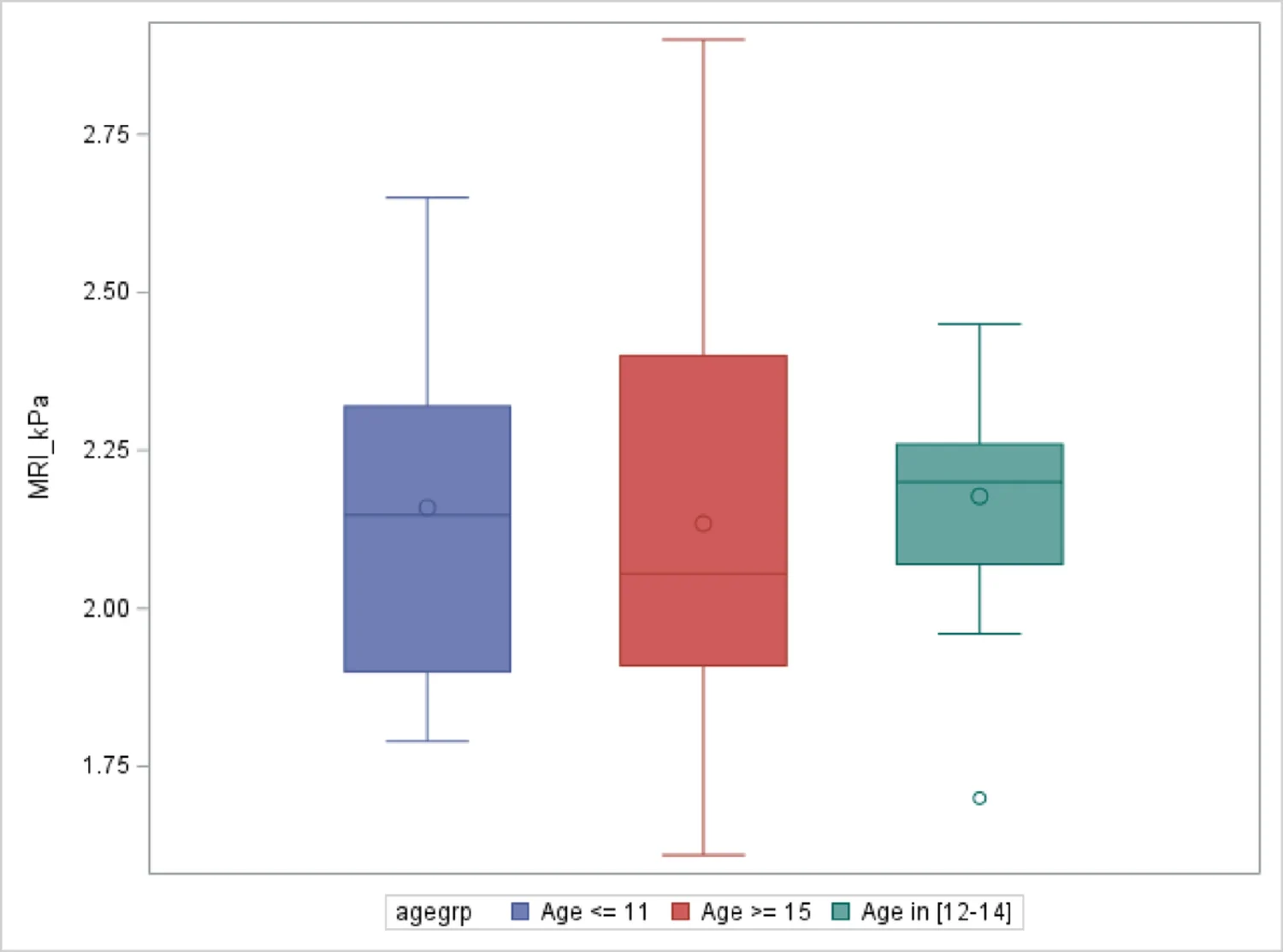
MRE liver stiffness (MRI_kPa) values by age in Box-plot

**Fig. 4:**
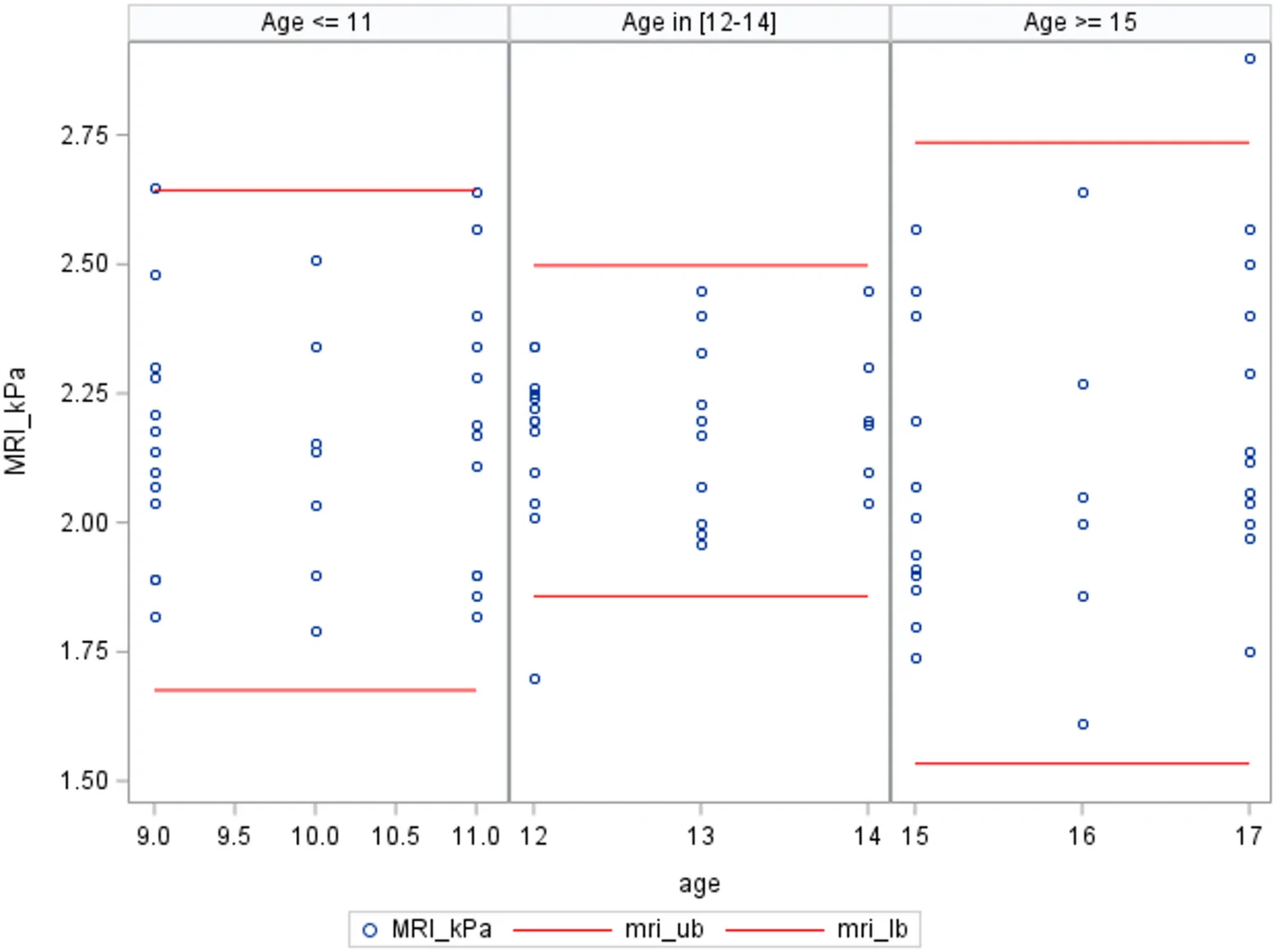
MRE stiffness reference interval (MRI_kPa) by ages, upper limit of normal (mri_ub) and lower limit of normal (mri_lb)

**Fig. 5:**
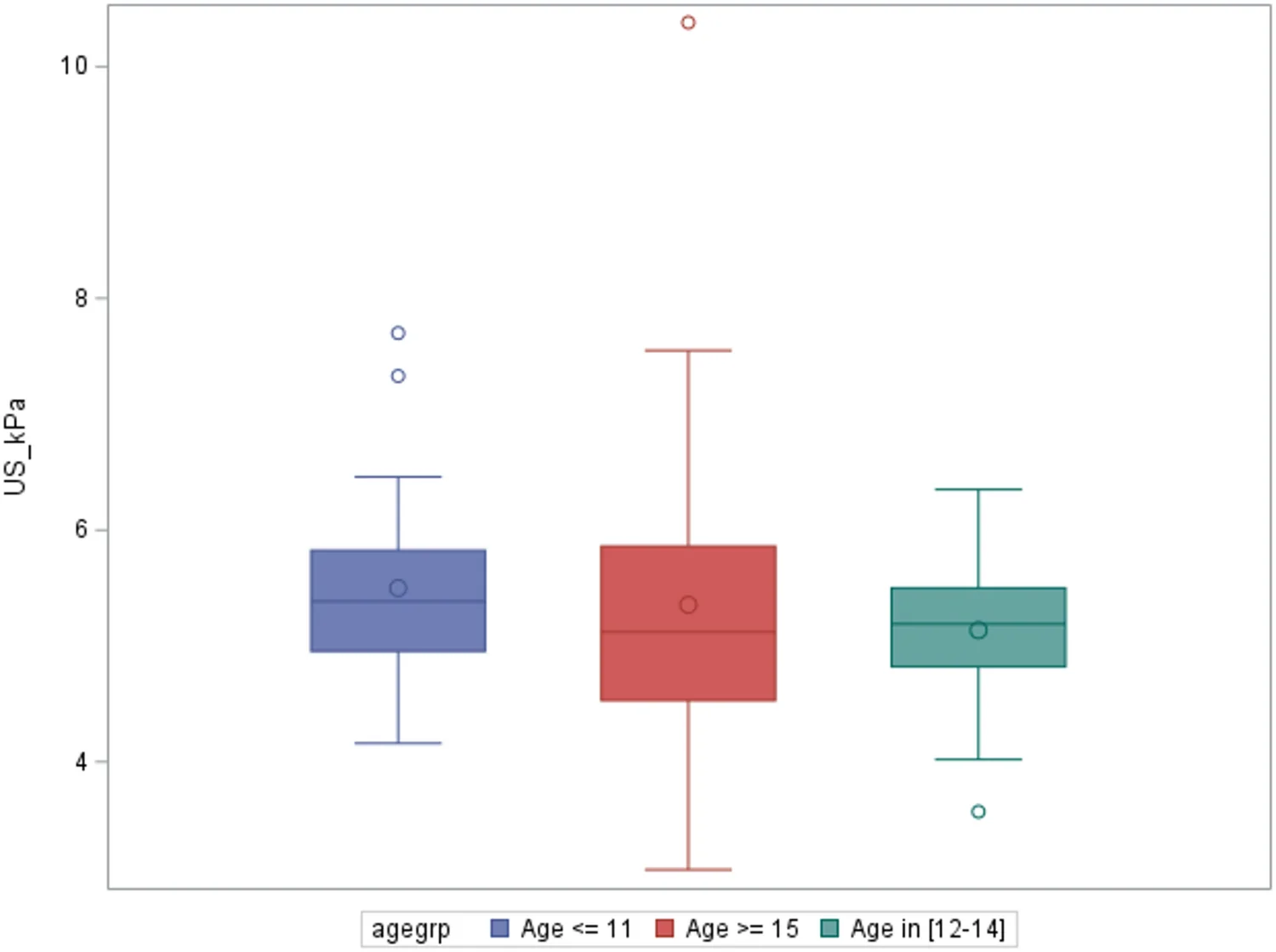
US elastography liver stiffness reference interval (US_kPa) values by age in Box-plot

**Fig. 6:**
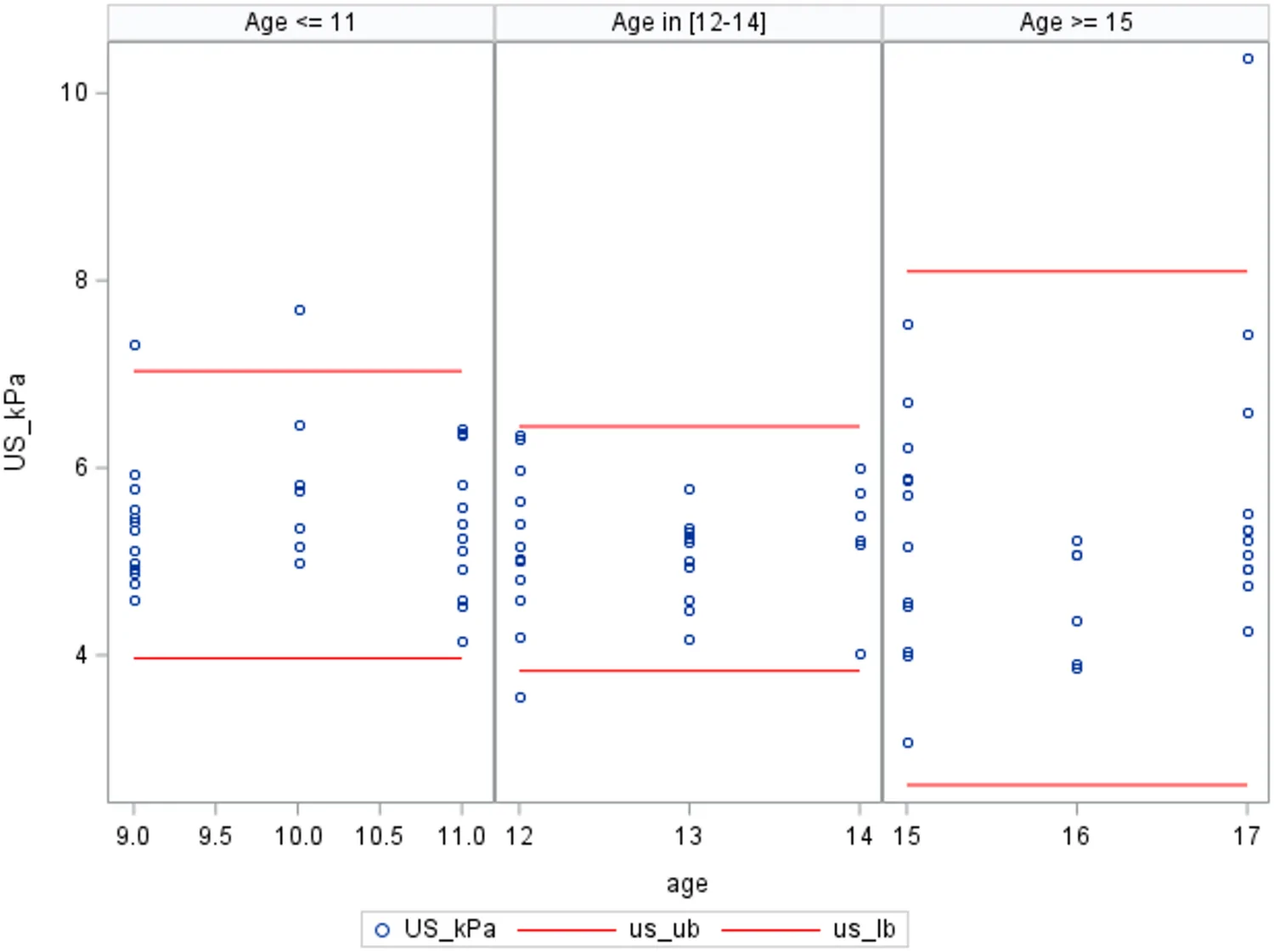
US stiffness reference interval (US_kPa) by ages, upper limit of normal (us_ub) and lower limit of normal (us_lb)

Liver stiffness did not differ significantly across age groups (9–11, 12–14, 15–17 years) for either MRE or 2D‑SWE (Table 4). Consistent with this finding, liver stiffness measurements were not significantly correlated with age (Table 3). Age‑specific mean liver stiffness values, standard deviations, and 95% reference intervals are presented in Table 4 and illustrated in Figs. 3–6; the 95% reference interval denotes mean ±1.96 SD. As a sensitivity analysis, we have also presented the normative ranges based on bootstapping estimates in Table 4.

**Table 2:** Liver MR-ultrasound elastography stiffness values by sex (F: Female, M: Male)

		n	Min	Median	Max	Mean	SD
MR elastography (kPa, *p* = 0.91)	F	47	1.61	2.17	2.90	2.16	0.25
	M	44	1.70	2.15	2.65	2.16	0.24
US elastography (kPa, *p* = 0.85)	F	47	3.07	5.17	10.38	5.36	1.15
	M	44	3.86	5.25	7.70	5.31	0.82

**Table 3:** Correlation between liver stiffness values and other variables (*ρ*: spearman correlation coefficient)

Variables	ρ, MR elastography	*p*	ρ, US elastography	*p*
Age	−0.02	0.82	−0.12	0.26
Height	−0.02	0.84	−0.11	0.32
Weight	−0.12	0.27	−0.18	0.093
Body-mass index	−0.28	0.0082	−0.22	0.039
MR fat fraction	−0.14	0.18	−0.12	0.25
R2*	0.03	0.75	−0.05	0.67
R2 water	0.03	0.77	0.02	0.84
ALP	0.00	0.98	−0.07	0.51
ALT	−0.13	0.22	−0.01	0.91
AST	0.01	0.93	0.16	0.13
GGT	−0.03	0.76	−0.16	0.12
Direct bilirubin	−0.15	0.15	−0.24	0.019
Total bilirubin	−0.13	0.20	−0.27	0.0092

**Table 4:** Normal liver stiffness values with a 95% reference interval calculated by the bootstrap method across age groups

		n	Mean	SD	LLN	ULN	Bootstrap LLN	Bootstrap ULN
MR elastography (kPa, *p* = 0.46)	Total patients	91	2.16	0.24	1.69	2.63	1.74	2.64
	9–11 age	32	2.16	0.25	1.67	2.65	1.79	2.65
	12–14 age	29	2.18	0.16	1.87	2.49	1.70	2.45
	15–17 age	30	2.13	0.31	1.52	2.74	1.61	2.90
US elastography (kPa, *p* = 0.28)	Total patients	91	5.34	1.00	3.38	7.30	3.86	7.55
	9–11 age	32	5.50	0.78	3.97	7.03	4.16	7.70
	12–14 age	29	5.14	0.67	3.83	6.45	3.57	6.35
	15–17 age	30	5.36	1.40	2.62	8.10	3.07	10.38

*n* total number of patients, *SD* standard deviation, *LLN* lower limit of normal, *ULN* upper limit of normal, *Bootstrap LLN* lower limit of normal estimated by bootstrap method, *Bootstrap ULN* upper limit of normal estimated by bootstrap method

## Discussion

In this prospective single-center study of clinically and biochemically healthy Turkish children aged 9–17 years, we established modality-specific liver stiffness reference intervals for same-day MRE and 2D-SWE examinations. Although multiple studies have reported pediatric normative stiffness values, comparative reference intervals acquired with both modalities on the same day in the same healthy cohort remain limited; therefore, the present work provides modality-specific reference intervals and inter-modality benchmarking information under standardized acquisition conditions. Importantly, these reference intervals are specific to the studied population and acquisition protocol and should not be extrapolated to other pediatric populations without validation.

Across the study cohort, liver stiffness did not differ by age group or sex for either modality. Mean stiffness values were 2.16 ± 0.24 kPa for MRE and 5.34 ± 1.00 kPa for 2D-SWE (95% reference intervals: mean ±1.96 SD). These MRE findings are broadly consistent with prior pediatric reports that generally describe values around 2.1–2.2 kPa and find limited associations with age, sex, or scanner characteristics [21, 22, 25, 26]. However, the literature remains inconsistent regarding comparisons between pediatric and adult reference values, with some studies suggesting higher stiffness in children and others lower [25]. This inconsistency, alongside evidence from experimental models showing age-related variations in soft-tissue stiffness, reinforces the need for pediatric-specific benchmarking to avoid misclassifying mild fibrosis [27, 28].

In contrast, 2D-SWE reference values show greater between-study variability, with published pediatric reports spanning a wider range and variably describing age-related trends, including in Turkish pediatric cohorts [29–33]. In this context, our 2D-SWE values fall within the range reported in prior Turkish healthy-cohort studies [30, 31] US-based stiffness measurements are also more sensitive to vendor, protocol, and operator factors; therefore, modality-specific and locally validated reference tables are essential when interpreting pediatric stiffness values, particularly for US-based techniques [11–13]. MRE has been reported to have higher diagnostic performance and fewer technical failures than US-based elastography techniques for the evaluation of hepatic fibrosis [34].

MRE and 2D-SWE values were positively associated in our cohort; however, correlation reflects association and does not establish interchangeability or technical agreement. Therefore, MRE and 2D-SWE measurements should be interpreted as modality-dependent. Clinicians should therefore avoid using MRE and 2D-SWE values interchangeably when applying reference thresholds or longitudinally monitoring individuals.

Exploratory analyses suggested a weakly negative correlation between BMI and liver stiffness for both modalities. In regression modeling, the BMI slope for MRE was considered clinically negligible, while BMI was not a statistically significant predictor of 2D-SWE stiffness.

Furthermore, associations between stiffness and laboratory/serologic measures were not emphasized because eligibility criteria restricted values to normal/nonreactive ranges. Consequently, these correlations are of limited clinical relevance in this healthy cohort and may introduce restricted-range bias; these analyses are best considered hypothesis-generating.

This study has limitations. First, it was performed at a single center with a moderate sample size, which may limit generalizability. Second, children younger than 8 years were not recruited due to limited feasibility of reliable breath-holding for MRE and potential confounding from variable respiratory cooperation, so reference data for younger children remain to be established. Third, participants with BMI percentiles < 10th or > 95th were excluded to reduce confounding; consequently, these reference intervals may not fully reflect real-world pediatric elastography referrals, which often include overweight or obese children. Fourth, histopathologic correlation was not available, as biopsy was not clinically indicated in healthy volunteers, despite biopsy remaining the reference standard for fibrosis assessment [2, 5–7] Fifth, elastography measurements may be vendor-, protocol-, and operator-dependent, particularly for US-based techniques, and we did not evaluate inter-operator or inter-reader reproducibility [11–13] Finally, external validation in independent cohorts (including other pediatric populations and protocols/vendors) was not performed; therefore, the proposed reference intervals should be applied outside the present setting only after appropriate validation. Multicenter studies across vendors and age ranges, ideally including reproducibility assessments and external validation, are warranted.

## Conclusion and recommendations

Same-day MRE and 2D-SWE provided modality-specific liver stiffness reference intervals in clinically and biochemically healthy Turkish children aged 9–17 years. These protocol- and population-specific values may support noninvasive pediatric assessment but should not be used interchangeably across modalities or applied to other settings without external validation, multicenter studies across vendors, broader BMI ranges, and younger ages are warranted.

## Data Availability

The datasets generated and/or analyzed during the current study are not publicly available due to patient confidentiality and ethical restrictions but are available from the corresponding author upon reasonable request.
